# Manipulating the nonlinearity of transition-metal dichalcogenide polaritons

**DOI:** 10.1038/s41377-023-01319-8

**Published:** 2023-11-21

**Authors:** Min-Soo Hwang, Hong-Gyu Park

**Affiliations:** Department of Physics and Astronomy, and Institute of Applied Physics, Seoul National University, Seoul, 08826 Republic of Korea

**Keywords:** Nonlinear optics, Polaritons

## Abstract

The lithographically designed potential wells in monolayer WS_2_ microcavities are utilized to manipulate nonlinear transition-metal dichalcogenide polaritons and enhance the polariton-reservoir interaction strength.

Polaritons in condensed matter systems, especially in semiconductor crystals, have been intensively studied. These quasiparticles are generated when an elementary excitation interacts strongly with light. Notably, the large exciton oscillator strength, which is closely associated with electron-hole pairs, has been successfully demonstrated in numerous systems, including planar microcavities, plasmonic cavities, and photonic crystals, exhibiting strong light-matter coupling^1^.

Nonlinearity in conventional semiconductor microcavities reveals unique and intriguing phenomena beyond the linear regime. In conjunction with novel photonic designs and devices, nonlinearity of polaritonic emissions enables the generation of quantized states^2^. However, its usage has been limited due to the requirement for cryogenic temperatures. Weak nonlinearities in polariton systems operating at room temperature impeded their practical implementation.

For example, the low binding energy of excitons in GaAs microcavities restricts their functionality to cryogenic temperatures^1–3^. Semiconductor-based microcavities are afflicted by a number of factors, including a short lifetime, rapid degradation, and weak interactions. These factors cause high cavity losses and broad exciton linewidths, resulting in room-temperature operation within the linear regime or nonlinearity to cryogenic temperatures. Therefore, room-temperature-operating materials with intrinsically strong nonlinearities and high exciton binding energies have received considerable attention.

Atomically thin transition metal dichalcogenides (TMDs) materials that confine the electric field to a small volume provide a viable platform for overcoming these limitations. TMD monolayers produce stable excitons at room temperature, exhibiting robust nonlinear behavior due to the enhanced Coulomb interaction^4^. For example, MoS_2_, MoSe_2_, WS_2_, and WSe_2_ monolayers have demonstrated remarkable higher-order susceptibility^2,3^.

The challenge of confining polaritons with TMDs has led to a new quantum phase in the study of quasiparticle manipulation. However, the majority of recent accomplishments rely on random perturbations of local deformations or voids in the samples. The presence of uncontrollable potential sites in previous research prompted further investigation into the localization of TMD polaritons. One example is the attempt to analyze the nonlinearity of exciton polaritons by combining distributed Bragg reflectors (DBRs) with TMDs in photonic device structures^5^. While it may appear more practical to use photonic DBRs to confine TMD polaritons, the intricate processes occurring within these open cavities present challenges in the nonlinear response of polaritons. Therefore, deterministic on-site localization of nonlinear polaritons has significant implications for functional polaritonic devices.

In the work newly published in *Light: Science & Applications*^6^, Xiong’s research group proposed a new technique for manipulating nonlinear polaritons with lithographically defined potential landscapes in monolayer WS_2_ microcavities (Fig. 1). The WS_2_ monolayer was transferred to the bottom DBR consisting of 16 pairs of SiO_2_/TiO_2_ layers. The mesa cavities were patterned by the polymethyl methacrylate (PMMA) spacer before the deposition of the top DBR. The sizes of the cylindrical-shaped mesa structures ranged from 6.5 μm to 2 μm, with an average depth of about 59 nm, which sufficiently exceeded the exciton linewidth. Angle-resolved photoluminescence spectroscopy was used to characterize the exciton-photon interaction of the mesa cavity under a continuous-wave laser at 532 nm.

**Fig. 1:**
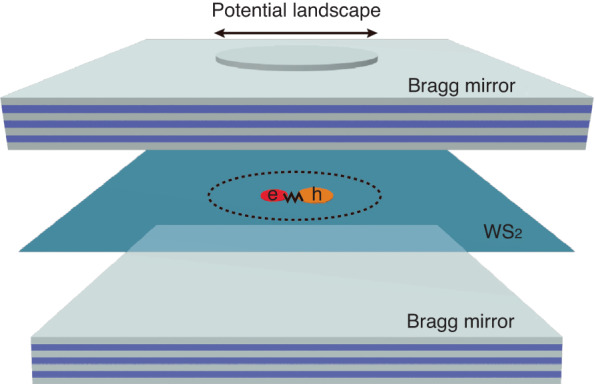
**Microcavity polaritons with a potential landscape**. A WS_2_ monolayer is sandwiched between two highly reflective distributed Bragg reflectors (DBRs). The artificial potential traps exciton polaritons in the cylindrical-shaped mesa structure

The strong coupling between excitons and cavity photons leads to the dispersion of anti-crossing behavior that splits into lower and upper polariton branches. The dispersion analyzed by a coupled oscillator model satisfies the criterion for strong coupling and confirms the formation of TMD polaritons. As the diameter of the trap decreases to 6.5 μm, 3 μm, and 2 μm, the in-plane momentum gradually expands and the states of *s*, *p*, and *d* energy bands are extracted. Based on the real-space image of the trapped polariton, the authors identified *s*, *p*, and *d* states corresponding to a cylindrically symmetric spot, a ring-shaped distribution, and a combined image composed of both the spot and ring-shaped distributions, respectively.

Next, the authors analyzed the polaritonic nonlinearity affected by spatial trapping, using the *s* state as a reference. The *s*-state emission shows a notable blueshift when the power is increased from 55 μW to 1002 μW. The nonlinear polariton-exciton interaction results in the energy shift being approximately linear to the exciton density, *ΔE* = *g*_*p–x*_*N*_*x*_, where *g*_*p–x*_ is the polariton-reservoir interaction constant proportional to the exciton fraction in the polariton states and *N*_*x*_ is the polariton density. Varying the size of the trap, the spatial localization of exciton-polaritons influences the discretization of photoluminescence dispersions and spatially confined patterns. All peak positions of *s* states are linearly shifted with increasing exciton density; however, the smaller the mesa cavity, the greater the peak shift.

The blueshifts of the peak position in different mesa sizes can also be plotted in relation to the exciton-photon detuning. These results demonstrate a nonlinear response to changes in trap size. At a detuning of –69 meV, the slope for the exciton density-dependent energy shift varies with the cylindrical-shaped mesa cavity sizes. This reflects that the wavefunction distribution of polariton, influenced by the trap sizes, determines the exciton-polariton spatial overlap and the interaction coefficient. At a detuning of –33 meV, *g*_*p–x*_ also increases dramatically as the trap size decreases. These findings indicate that the polariton-exciton interactions can be significantly enhanced by increasing spatial confinement.

In conclusion, the integration of room-temperature excitonic materials, such as TMDs, perovskites, and organic semiconductors, with micro/nano cavities for practical applications is ongoing. This technique aims to eliminate the majority of losses caused by the dephasing mechanism. Nonetheless, the spatial limitations between the electric field profile and the exciton wavefunction pose additional challenges when coupling photonic systems with exciton materials. In several experimental implementations to date, including this work, the combination of TMD monolayers and patterned microcavities continues to attempt to overcome the spatial limitations, while boosting the efficiency of nonlinear interactions^6–8^. These efforts to develop nonlinear polaritonic devices can ultimately yield coherent optical sources, multi-emitter systems, and polariton emitters that are electrically injected. Nonlinear optical processes will play a central role in establishing quantum networks, which are profoundly essential for signal processing in on-chip photonic devices.
